# Bioengineering adult human heart tissue: How close are we?

**DOI:** 10.1063/1.5070106

**Published:** 2019-03-14

**Authors:** Richard J. Mills, James E. Hudson

**Affiliations:** QIMR Berghofer Medical Research Institute, Brisbane, Queensland 4006, Australia

## Abstract

Human pluripotent stem cells (hPSCs) have extensive applications in fundamental biology, regenerative medicine, disease modelling, and drug discovery/toxicology. Whilst large numbers of cardiomyocytes can be generated from hPSCs, extensive characterization has revealed that they have immature cardiac properties. This has raised potential concerns over their usefulness for many applications and has led to the pursuit of driving maturation of hPSC-cardiomyocytes. Currently, the best approach for driving maturity is the use of tissue engineering to generate highly functional three-dimensional heart tissue. Although we have made significant progress in this area, we have still not generated heart tissue that fully recapitulates all the properties of an adult heart. Deciphering the processes driving cardiomyocyte maturation will be instrumental in uncovering the mechanisms that govern optimal heart function and identifying new therapeutic targets for heart disease.

## INTRODUCTION

The maintenance and expansion of embryonic stem cells in a pluripotent state^1^ have been key to enabling the production of a number of different human cell types, in particular, those that were traditionally hard to expand and culture [e.g., cardiomyocytes (CMs)]. Additionally, the discovery that differentiated adult cells could be reprogrammed back to a pluripotent state using defined factors, first in mouse cells^2^ and then in human cells,^3^ now enables the generation of pluripotent stem cells from nearly any patient. These seminal discoveries, together with advanced directed differentiation protocols,^4^ have led to widespread international use of human pluripotent stem cell (hPSC)-derived cells in both academic and industry led biomedical research.

**FIG. 1.**
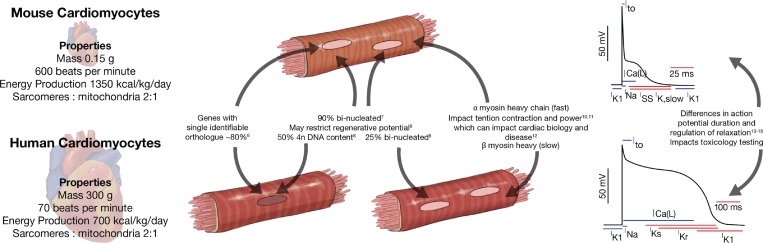
Differences in characteristics of mouse versus human cardiomyocytes.

Human cardiomyocytes have many distinct features compared to rodent cardiomyocytes, which have been used as the model system of choice for decades (Fig. 1). To gain a closer representation of human hearts, larger animal models such as rabbits, cats, dogs, sheep, pigs, and monkeys have also been utilized throughout the literature for physiological studies. However, ethical considerations, longer time frames, and higher costs are inhibitory to the widespread use of these larger animal models for routine studies. Therefore, hPSC-derived cardiomyocytes (hPSC-CM) have become an integral part of biological studies and drug discovery in the hope that they may help facilitate translation of research to the clinic.

Many physiological properties such as the size, contraction rate, cardiac output, and metabolism differ between human and mouse hearts.^5^ Furthermore, around 20% of the protein coding genes in the mouse do not have a single human orthologue.^6^ These differences give rise to many disparities in the biology of the cardiomyocytes themselves. For example, mouse cardiomyocytes are predominantly bi-nucleated^7^ compared to human cardiomyocytes being predominantly polypoloid.^8^ This may have substantial biological significance, as recent studies suggest that that the fraction of binucleated cardiomyocytes has a profound impact on the regenerative potential of the heart.^9^ Additionally, there are differences in which myosin heavy chain isoforms are predominantly expressed. This impacts how tension and power are generated during contraction, affecting the mechanisms underpinning cardiac disease and function.^10–12^ Finally, the action potential of mouse and human cardiomyocytes is very different. In fact, this is not just a mouse versus human difference, as the action potential duration tends to scale with the animal size to maintain cardiac output.^13–16^ Interestingly, many of these differences between mouse and human cardiomyocytes observed *in vivo* are also observed in CM when mouse pluripotent stem cell and hPSC-CM are compared.^17–20^ This indicates that while some parameters are dictated by animal physiology, many are also inherently encoded in the genome.

### Directed differentiation of hPSC into cardiomyocytes

Decades of research in developmental biology have been essential for the generation of hPSC-CM. This research provided the developmental blueprint to differentiate hPSCs into cardiomyocytes. Originally, non-directed spontaneous differentiation approaches were used (through withdrawal of stem cell maintenance factors). However, these protocols are inconsistent and generate very low percentages of cardiomyocytes. Over the past 15 years, directed differentiation protocols have greatly advanced and have now improved the efficiency of cardiac differentiation to yield greater than 95%. Directed differentiation protocols enable robust and reproducible differentiation and follow known developmental stimuli such as endoderm co-culture,^21^ growth factor signaling,^22–26^ and biological pathway manipulation through small molecules.^27,28^ It is even possible to now purchase hPSC-CM commercially or buy differentiation kits, making hPSC-CM more widely available. These protocols tend to generate predominately ventricular cardiomyocytes although protocols have been recently developed to also efficiently produce nodal^29^ and atrial cardiomyocyte^30,31^ sub-types. Therefore, the three major cardiomyocyte sub-types are now readily accessible for biomedical research.

### Maturity of hPSC-CM

hPSC-CMs cultured in 2D have many of the key features of their *in vivo* counterparts. This makes them a very useful and predictive model for many applications including studying hypertrophy, electrophysiology, drug toxicity and discovery, and fundamental biology (reviewed in Ref. 32) However, due to the relative immaturity of 2D hPSC-CM cultures, they have failed as a model for some applications (reviewed in Ref. 33). Responses inconsistent with adult hearts have been observed in studies on: sarcomeric cardiomyopathies such as TITIN mutations associated with dilated cardiomyopathy,^34^ metabolic syndromes such as mutations in tafazzin,^35^ cell cycle re-entry where 2D cells respond to mitogens,^36^ and discovery of bona fide inotropes.^37,38^ Therefore, induction of hPSC-CM maturation is required to improve modelling capabilities of hPSC-CM.

### Why are hPSC-CMs not mature?

Why has maturity similar to that of the adult human heart not yet been achieved? Directed differentiation of hPSC into CM has been very successful, as it builds on decades of research into developmental biology. This research identified the processes that govern the specification and differentiation of early mesoderm and subsequently cardiomyocytes *in vivo*, which could then be applied to hPSC-CM differentiation. However, the processes known to govern postnatal maturation of the heart are limited and the molecular mechanisms of cardiac maturation remain to be deciphered.

Typical 2D cultures of hPSC-CM cultures fail to reach adult transcriptional maturity even after prolonged culture times (i.e., 1 year).^39,40^ This indicates that either (1) the development of a fully differentiated adult cardiomyocyte is genetically programmed and may take up to 20 years^8^ or (2) we have not yet found the key drivers and molecule mechanisms driving adult maturation. Interestingly, some maturation processes such as the cell cycle exit and loss of regeneration capacity occur in a similar time window after birth in both small^41,42^ and large mammals.^43,44^ This therefore indicates that some environmental factors are key upstream drivers of maturation and maturation is not solely a genetically timed process. Understanding the environmental drivers of maturation and how they impact cardiomyocytes may therefore help us drive adult maturation in hPSC-CM.

### What is maturation?

hPSC-CM maturation is not defined by a single property nor is it driven by a single stimulus. There are a wide array of properties which need to be assessed, covering many aspects of cardiac biology and function (Table I). These require a wide array of assays which need to be performed to assess the different aspects of maturation.^39,40,45,46^ This is an important point, as adult maturation cannot be stated unless all these properties have been obtained by the hPSC-CM. As Table I highlights, cardiomyocyte properties that change during postnatal heart maturation gear them towards a highly functional and efficient cell. Hence, the key measures that encapsulate multiple aspects of cardiomyocyte maturity are related to their functional properties [Table II (Refs. 8, 9, 36, 39, 40, and 47–64)].

**TABLE I.** Properties of immature versus mature cardiomyocytes. F-S: Frank-Starling mechanism where increased sarcomere length leads to increased force of contraction.

Property	Immature 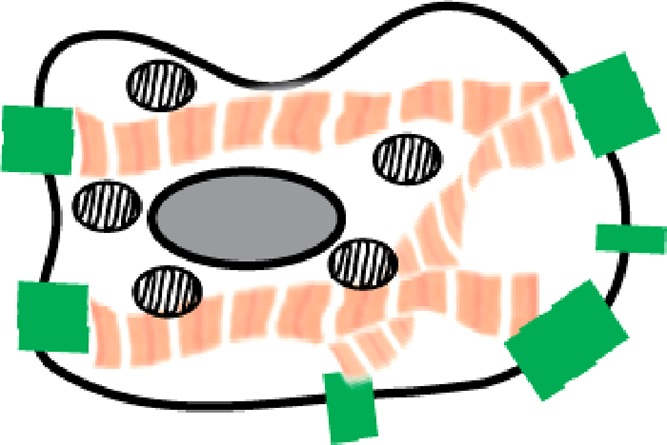	Mature 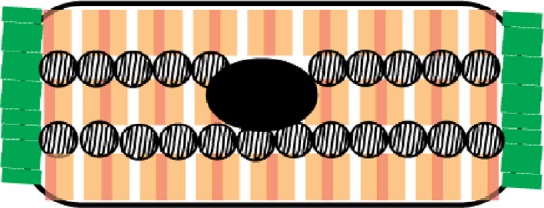	Impact with maturation
Sarcomeres	Irregular, 10% volume^47^	Organized, 40% volume^47^	↑Force generation
	1.8 *μ*m sarcomere spacing^48^	2.2 *μ*m sarcomere spacing^48^	↑Force generation (F-S)
	Proteins are fetal isoforms^40^	Proteins are adult isoforms^40^	Power generation,^10–12^ ↑stiffness,^40,50^ signalling^51^
Calcium handling	Immature^48,52^	Mature^48,52^	↑Calcium amplitude^48,52^
			↑Force generation
			↑Faster activation and decay^48,52^
T-tubule system	Poorly developed and organized^53^	Highly developed and organized^54^	↑Synchronous and efficient calcium activation throughout the cell^54^
Ion channel expression	Fetal isoform of I_Na_^55^	Adult isoform of I_Na_^55^	↑Upstroke velocity^55^
	Low expression of I_K_^40^	High expression of I_K_^40^	↓Resting membrane potential^56^
Gap junction organization	Circumferential^57,58^	Polarized to ends (at intercalated discs)^57,58^	↑Anisotropic conduction velocity
			↑Anisotropic force generation
Metabolism	Glycolytic 10% mitochondria^59^	Oxidative phosphorylation 30% mitochondria^60,61^	↑ATP production^60,61^
			↑Oxygen usage^59–61^
			↑Fatty acids > glucose^60,61^
Cell Cycle	Mitogens drive proliferation^36,40^	Mitogens drive hypertrophy^62^	↑Cardiomyocyte size^62^
			↓Regenerative potential^62^
Nucleus/DNA content	Mono-nucleated monoploid^8^	25% binucleated	↓Regenerative potential?^9^
		50% mono-nucleated polyploidy	
		25% mono-nucleated monoploid^8^	
ECM binding	β1 integrin collagen I/fibronectin^63,64^	Laminin/basement membrane^63,64^	↓Proliferation^63,64^

**TABLE II.** Key measurements of maturity. Properties and references are outlined in Table I.

**Contractile force and kinetics**
Mature cardiomyocytes produce higher forces and have faster upstroke and decay rates. Changes in sarcomere proteins and their organization, calcium handling, t-tubule organization, ion channel expression, and ECM binding all influence contractile force and kinetics.
**Response to adrenergic stimulation**
In mature cardiomyocytes, there is a chronotropic, lusitropic, and inotropic response to adrenergic stimulation. Changes in sarcomere proteins and their organization, calcium handling, t-tubule organization, and ion channel expression all influence this response.
**Increased force-frequency relationship**
Mature cardiomyocytes increase force as their rate increases (positive staircase). This is heavily influenced by the proteins involved in calcium handling and cellular compartment organization.
**Conduction velocity**
Electrical conduction velocity is faster in mature cardiomyocytes. This is regulated by cardiomyocyte coupling via cell-cell connections and gap junctions and by ion channel expression and regulation. Additionally, resting membrane potential of the cardiomyocytes influences this property.
**Transcriptome**
The adult cardiomyocyte transcriptome is distinct from an immature cardiomyocyte. There are extensive changes in expression of sarcomeric protein isoforms, metabolic genes, and cell cycle genes during this process.^65^ The transcriptome can be potentially used as an unbiased holistic measure of maturity^40,66^ but should be used in combination with functional assays.
**Metabolism**
During maturation, there is a switch from glycolysis to fatty acid metabolism. This facilitates a high metabolic capacity and increased mitochondrial biogenesis.

### Bioengineering for maturation

As Table I shows, many functional properties that characterize maturation are the result of the highly controlled organization and compartmentalization of sub-cellular structures. This includes sarcomere alignment, t-tubule organization, formation of sarcoplasmic reticulum adjacent to the t-tubules and sarcomeres, polarized cardiomyocyte-cardiomyocyte junctions to couple both tension and ion flux, organized mitochondria adjacent to the sarcomeres, and cell-cell interactions between different cell types. One way to promote these features is to create a bioengineered environment, allowing the formation of a complex 3D tissue and thus recapitulating *in vivo* organ-like structures.^67^ Cells *in vivo* behave very differently from their *in vitro* counterparts on stiff, flat, un-patterned 2D substrates.^68^ Therefore, researchers have used bioengineering to produce cell culture substrates to help mature some properties of cardiomyocytes, including soft hydrogels,^69,70^ substrate patterning,^71–74^ or flexible substrates that can deform.^74–76^ While some of these approaches have generated constructs that achieve high forces of contraction indicative of maturation (up to 10 mN/mm^2^), a wider array of properties resembling an adult heart have only been shown thus far within 3D engineered heart tissue (EHT) formats.^77–79^

EHT has been used for decades to create highly functional heart muscle. EHT was originally derived from isolated chicken or neonatal rat heart cells and cultured inside collagen I/Matrigel hydrogels.^80,81^ Later, fibrin/thrombin based methods have also been used to form EHT.^82,83^ It is apparent that the most important factor for the extracellular substrate is the ability of the cells to interact with the matrix. By using native ECM such as collagen I/Matrigel/fibrin, the cells can: (1) produce their own matrix which interlocks with these matrices, (2) secrete matrix metalloproteinases to re-arrange the matrix, and (3) bind the matrix for migration, tension generation, and tissue condensation. In addition, it has been shown that co-cultures of different cell types are required in EHT for optimal cardiac function.^24,39,84–86^ Having a 3D environment based on native matrices enables self-organization of cell-types into *in vivo*-like cardiac organization,^40^ thus promoting effective cell-cell communication.

In addition to cellular organization, the EHT approach enables precise mechanical loading of the sarcomeres. In the native heart, most of the mechanical tension is actually imposed on the sarcomeric giant protein TITIN at physiological strains.^87^ Having a hydrogel full of interconnected cardiac cells is one of the best ways to achieve sarcomeric loading *in vitro*. This may be an important step for maturation, as tension through cardiomyocyte-substrate binding in stiff 2D environments imposes different biological effects and may actually keep the cardiomyocytes in an immature state^63^ or impose disease-like states.^88^ This is also a key consideration for the design of synthetic biomaterials for cardiac tissue engineering, as a stiff polymer or an environment preventing mechanical loading through intercalated discs may be more representative of a 2D rather than a 3D environment.

A variety of different loading regimes have been utilized in the EHT format. Auxotonic mechanical loading, rather than static stretching or phasic stretching, is the most physiological means of EHT mechanical loading and results in enhanced functionality.^89–91^ This is not surprising as myocardial tissue is inherently designed to respond to mechanical loading for proper heart function. For example, the Frank-Starling mechanism whereby increased preload results in increased force of contraction normalizes beat-to-beat variations in ventricular filling. It is therefore paramount that bioengineering applications carefully consider mechanical loading regimes, as non-physiological loading may have considerable consequences on signaling^92^ (Table I) and has even been used to model heart disease in EHT.^90,93^

Overall, the EHT approach has many advantages in creating the most accurate model of heart tissue *in vitro*. The application of auxotonic mechanical loading and pacing^77,79,94^ has enabled the production of adult maturity in EHT derived from neonatal rat hearts. Therefore, this approach may also be one of the best methods to achieve adult maturation in hPSC-CM.

### Maturation of hPSC-CM

Advanced maturation of hPSC derived-EHT has been achieved by optimizing multi-cellularity and mechanical loading and pacing [Table III (Refs. 20, 23, 39, 40, 45, 46, 65, 93, and 95–107)]. A key finding in multiple studies is that these stimuli drive an increase in EHT function via maturation of the attributes required for optimal cardiac function (see Table I). However, the goal of full adult maturity has not yet been achieved using these approaches, potentially because we do not yet understand all the key drivers and molecular mechanisms of cardiac maturation.

**TABLE III.** Properties of EHT benchmarked against 2D culture and adult hearts. Note: Parameters were selected based on analyses of the function (Table II) and those that were measured in the majority of studies. #Tissue produced by the Eschenhagen Lab are more diffuse than other formats, and the functionality seems to be low; but the cardiomyocytes on a per cell basis display a high degree of maturation and are highly functional.

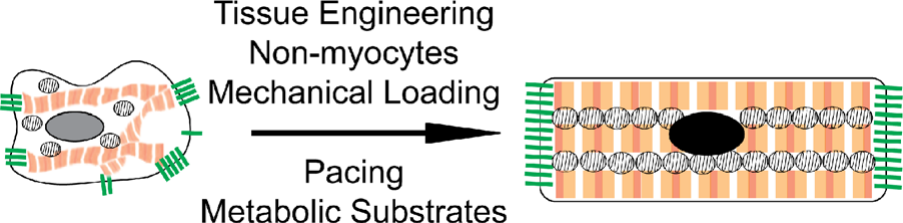				
	Approach	Force (mN/mm^2^)	Isoprenaline force increase (% at EC_50_ Ca^2+^)	Mechanical loading regime
**Adult heart**		25^95^	200^96^	Sarcomeres loaded
				Auxotonic
				Preload: ventricular filling
				Afterload: systolic blood pressure
**2D cardiomyocytes**		0.25–0.5^101^	Inconsistent	Dependent on the substrate
				Tissue culture plastic has an elastic modulus 100 000 times the heart
				Most mechanical loading through ECM-integrin rather than sarcomere loading
**Bursac Lab**	Fibrin		70	Loaded 3D gel facilitating sarcomeric loading
Zhang *et al.*^45^	Stromal cells			Auxotonic
Jackman *et al.*^98^	± Medium convection	−12/+23		Preload: endogenous cell tension
Shadrin *et al.*^102^				Afterload: undefined–Velcro frame
**Zimmermann Lab**	Collagen I	6.2	80–90	Loaded 3D gel facilitating sarcomeric loading
Tiburcy *et al.*^39^	Stromal cells			Auxotonic
				Preload: 10% strain and endogenous cell tension
				Afterload: controlled by elastic posts
**Vunjak-Novakovic Lab**	Fibrin	4	75	Loaded 3D gel facilitating sarcomeric loading
Ronaldson-Bouchard *et al.*^46^	Stromal cells			Auxotonic
	Pacing			Preload: endogenous cell tension
				Afterload: controlled by elastic posts
**Sniadecki/Murry Lab**	Fibrin	0.4		Loaded 3D gel facilitating sarcomeric loading
Leonard *et al.*^93^	Stromal cells			Auxotonic
				Preload: endogenous cell tension
				Afterload: controlled by elastic posts
**Murry Lab**	Collagen I/Geltrex	1.3		Loaded 3D gel facilitating sarcomeric loading
Ruan *et al.*^103^	Stromal cells			Static loading
	Pacing			Preload: endogenous cell tension
				Afterload: dependent on force generation
**Conklin/Healy Lab**	No ECM	4	∼50	Cardiomycyte/stromal cell mixture adhered to 2D substrate at either end
Huebsch *et al.*^104^	Stromal cells			Static loading
				Preload: endogenous cell tension
				Afterload: dependent on force generation
**Hudson/Porrello Lab**	Collagen I/Matrigel			Loaded 3D gel facilitating sarcomeric loading
Voges *et al.*^23^	Stromal cells			Auxotonic
Mills *et al.*^107^	±Metabolic maturation	7	50	Preload: endogenous cell tension
Mills *et al.*^40^				Afterload: controlled by elastic posts
**Eschanhagen/Hansen Lab**	Fibrin	0.06#	41	Loaded 3D gel facilitating sarcomeric loading
Schaaf *et al.*^105^	±Stromal cells			Auxotonic
Hirt *et al.*^94^	±Pacing			Preload: endogenous cell tension
Mannhardt *et al.*^20^				Afterload: controlled by elastic posts
Ulmer *et al.*^106^				

In addition to the aforementioned stimuli, metabolism has also been recently identified as a major driver of maturation (Table III). In the EHT environment^40,106^ or even in prolonged 2D culture^59,100^ (but not to the same extent^106^), hPSC-CMs increase their capacity for energy production via increased production of mitochondria and in-turn oxidative phosphorylation capacity. This increased respiratory capacity seems to be induced via a contraction-tension based mechanism,^106^ but further work is required to elucidate the detailed molecular mechanisms involved. Further downstream, PGC-1α has been identified to be likely involved in coordinating these metabolic changes.^59,106^ While oxidative phosphorylation can increase in the EHT environment with mechanical loading, switching metabolic substrates from glucose/carbohydrates to fatty acids significantly alters cellular metabolism towards oxidative phosphorylation and promotes further maturation.^40,100^ Exactly how this drives maturation remains to be deciphered, but it is known to be associated with a DNA damage response and the repression of Wnt-β-catenin and yes-associated protein 1/tafazzin signaling.^40^ Determining the mechanisms behind this maturation process may help us further advance maturation of hPSC-CM.

### Similarities to *in vivo* maturation

In hPSC-CM studies so far, the drivers of maturation are consistent with environmental changes that occur *in vivo*. In the early postnatal window, there is a shift in many cardiac parameters and recapitulating some of these in hPSC-CM has been shown to result in maturation (Table III). However, there are a considerable number of changes in the postnatal environment (Table IV), and it is still unclear to what extend each of them influences maturation of the heart. Additionally, there are considerable changes in multiple organs during postnatal development, and therefore, inter-organ communication and even interactions between the microbiome and the host^108^ may be important. The study of these processes not only is pertinent to cardiac maturation but may also be critical of other organ types, as many hPSC-derived cell types are considered “immature.”

**TABLE IV.** Factors changing in the postnatal environment (non-exhaustive)

**Oxygen tension**
Before birth, the arterial oxygen tension is only $P_{O_{2}}$ = 30 mmHg and increases around 3-fold after birth to 100 mm Hg. This has a large impact on metabolism, and the heart transitions from a relatively hypoxic environment where glycolysis is the primary form of energy production to oxidative phosphorylation after birth.^97,109^ This also creates reactive oxygen species affecting cardiac maturation and additionally has widespread consequences throughout the body.^110^
**Catecholamines (norepinephrine, epinephrine, and dopamine)**
There is a release of catecholamines following birth to: (1) increase cardiac output, (2) stimulate gluconeogenesis and glycogenolysis in the liver, (3) release free fatty acids, and (4) regulate blood pressure.^111^
**Metabolic substrates**
Energy production undergoes a switch, as there is a shift from a carbohydrate based to a fatty acid dominated metabolism the newborn starts feeding on breast milk. This induces widespread physiological adaptations such as induction of mitochondrial biogenesis, gluconeogenesis, glycogenolysis, and ketogenesis in the liver to supply other metabolic substrates.^112^
**Serum proteome**
The serum proteome undergoes major changes after birth.^113^ Considerable work is required to determine the impact of these changes and which factors are important for cardiac maturation.
**Cellular composition**
The cellular composition in the myocardium changes during postnatal maturation. There are varying estimates in the percentage of cells that are cardiomyocytes; however, the general consensus is that the fraction of cardiomyocytes decreases during the maturation period.^114–116^ The stromal fraction also changes during postnatal maturation;^117^ in some papers, this has been shown to change from 52:41:6.5 to 52:25:20 endothelial cells:fibroblasts:leukocytes during postnatal maturation and is thus a major change in the fibroblast to leukocyte/macrophage ratio.^65^ It is still unclear how this influences cardiac maturation and how the different cell populations interact with each other, but there is increasing evidence of complex interplay between the cell types.^118^

### Deciphering maturation programs is important for cardiac disease and regeneration

Cardiac dysfunction during disease has many facets and can be attributed to many different processes.^119,120^ It is well established that the neonatal heart is better adapted to many insults in comparison to the adult heart^121^ and can functionally regenerate after injury.^41–44^ Therefore, understanding the maturation process may also lead to the understanding of how the adult heart becomes more susceptible to injury and thus unlock new therapeutic targets for disease. It has been a central dogma for decades that there is a reversion to a fetal phenotype in many disease states, including glycolysis, expression of fetal cardiac proteins, and a growth program (note: hypertrophy not hyperplasia). Reversion of some processes can be cardiac protective, but others can be detrimental. Given the reversion of some properties but not others in disease,^65^ it is critical that we gain a better understanding of the maturation process so that we can determine the most beneficial adaptions to be exploited as therapeutics.

### Application of mature hPSC-CM

Whilst we have not yet produced hPSC-CMs that are equivalent to adult cardiomyocytes, they are still very useful in a wide array of academic and industry based applications, including fundamental science, cell therapy, disease modelling, and drug discovery studies. Furthermore, it should be highlighted that adult hPSC-CM may not be the optimal maturity stage for some applications. For example, for cell therapy applications, engraftment and proliferation following implantation are improved if relatively immature hPSC-CMs are used.^122,123^ On the other hand, more mature hPSC-CM will be essential for modelling diseases such as Barth syndrome where metabolically mature cardiomyocytes are required.^35^ Therefore, the bioengineering approach used for different applications may vary considerably (as they currently do), and the optimal bioengineered platform needs to be carefully considered for each specific application.

Scaling of EHT for different applications is also an important consideration. Production and banking of billions of hPSC-CM are now achievable and have already been implemented by some academic labs and within industry. This topic has many aspects requiring consideration and has been extensively reviewed.^124^ More pertinent to this perspective is the scaling of EHT technologies, especially as culture formats become more and more complex and technically challenging to fabricate. It should also be noted that different applications require different scales. Miniaturization of hPSC-CM^36^ or EHT^40^ cultures is required for large-scale drug discovery or biological screening. Conversely, EHT patches for heart regeneration require larger formats.^39^ When scaling EHT, there are a number of aspects which may require considerable engineering for optimization. Two of the most important considerations are as follows.

#### Complexity

For all applications, it is essential that the EHT approach used is consistent and reproducible. Therefore, when introducing more complexity into EHT systems, such as pacing, mechanical loading, metabolic substrates, or greater complexities in cell composition, increased quality control is required. When using EHT as a model system, electrically paced EHT formats require that all tissues are paced at the same rate with the same current. In low throughput systems, this is easily managed; however, this becomes increasingly difficult in higher throughput systems where potentially 1000s of miniaturized EHTs are cultured. Furthermore, incorporating more complexity into larger implantable EHT results in additional costs for both the fabrication of the EHT and the additional impact to the Good Manufacturing Practice production pipelines. Overall, additional EHT complexity to enhance maturity requires careful consideration of cost versus benefit when scaling the technology for drug screening or regenerative medicine applications.

#### Size

The heart is the most metabolically active organ and consumes roughly 20 times its weight in adenosine triphosphate (ATP) daily.^125^ This has huge consequences on EHT design to ensure sufficient oxygen and nutrient supply. In miniaturized formats where the cell layers are only ∼60 *μ*m (a few cell layers thick) ,^40^ metabolite or oxygen diffusion is not limited. However, larger formats require strategies to improve supply in order to avoid formation of a necrotic core. A variety of techniques have been successful in achieving this, including having more diffuse muscle bundles,^94^ incorporating perfused endothelial tubes,^126^ or having dynamic culture vessels to increase media convection.^98^

## CONCLUSION—HOW CLOSE ARE WE?

The holy-grail of cardiac bioengineering is the production of fully mature adult hPSC-CM, which is also a pursuit for many other organ and cell types.^127,128^ Driving full adult hPSC-CM maturation will not only result in a better model for many applications but also decipher the poorly understood maturation process of the heart. There has been great progress in this area, and bioengineered models have been instrumental for advanced maturation of many hPSC-CM properties. However, further work is required to progress maturation to an adult state, which will require determination of how the postnatal environment drives maturation. Due to the large number of postnatal physiological changes of the heart, it is likely a multi-faceted approach that mimics mechanical loading, pacing, metabolic substrates, and cellular-composition of the adult heart will be required to drive adult maturation of EHT. How each of these factors influence cardiac maturation and their underpinning molecular mechanisms require detailed follow-up investigations. Understanding of these processes will enable not only the generation of more mature hPSC-CM cultures but also the understanding of how heart biology and function are governed, which in itself may lead to novel therapeutic targets.
